# Vaginal pH and Microbicidal Lactic Acid When Lactobacilli Dominate the Microbiota

**DOI:** 10.1371/journal.pone.0080074

**Published:** 2013-11-06

**Authors:** Deirdre E. O’Hanlon, Thomas R. Moench, Richard A. Cone

**Affiliations:** 1 Thomas C. Jenkins Department of Biophysics, Johns Hopkins University, Baltimore, Maryland, United States of America; 2 ReProtect Inc., Baltimore, Maryland, United States of America; Rush University, United States of America

## Abstract

Lactic acid at sufficiently acidic pH is a potent microbicide, and lactic acid produced by vaginal lactobacilli may help protect against reproductive tract infections. However, previous observations likely underestimated healthy vaginal acidity and total lactate concentration since they failed to exclude women without a lactobacillus-dominated vaginal microbiota, and also did not account for the high carbon dioxide, low oxygen environment of the vagina. Fifty-six women with low (0-3) Nugent scores (indicating a lactobacillus-dominated vaginal microbiota) and no symptoms of reproductive tract disease or infection, provided a total of 64 cervicovaginal fluid samples using a collection method that avoided the need for sample dilution and rigorously minimized aerobic exposure. The pH of samples was measured by microelectrode immediately after collection and under a physiological vaginal concentration of CO2. Commercial enzymatic assays of total lactate and total acetate concentrations were validated for use in CVF, and compared to the more usual HPLC method. The average pH of the CVF samples was 3.5 ± 0.3 (mean ± SD), range 2.8-4.2, and the average total lactate was 1.0% ± 0.2% w/v; this is a five-fold higher average hydrogen ion concentration (lower pH) and a fivefold higher total lactate concentration than in the prior literature. The microbicidal form of lactic acid (protonated lactic acid) was therefore eleven-fold more concentrated, and a markedly more potent microbicide, than indicated by prior research. This suggests that when lactobacilli dominate the vaginal microbiota, women have significantly more lactic acid-mediated protection against infections than currently believed. Our results invite further evaluations of the prophylactic and therapeutic actions of vaginal lactic acid, whether provided in situ by endogenous lactobacilli, by probiotic lactobacilli, or by products that reinforce vaginal lactic acid.

## Introduction

Low Nugent scores (0-3) indicate lactobacillus-morphotype-dominated (“LMD”) vaginal microbiota [1]. LMD microbiotas are strongly associated with reduced risks of infections by reproductive tract pathogens, including HIV-1 [2,3], HSV-2 [4], *Trichomonas vaginalis* [5], *Neisseria gonorrhoeae* and *Chlamydia trachomatis* [6], as well as the multiple species of bacteria associated with bacterial vaginosis (BV) [7,8]. In addition, HIV-1 RNA is reduced in the cervicovaginal fluids from HIV^+^ women that have high concentrations of lactobacilli [9,10] and low Nugent scores [11].

Lactobacilli acidify the vagina with lactic acid [12,13], and some can also produce hydrogen peroxide under permissive and artificial laboratory conditions [14]. These ‘peroxide-producers’ are associated with a reduced incidence of BV [15,16] and some reproductive tract infections [17,18], compared to ‘non-producing’ lactobacilli. Under permissive *in vitro* conditions, H_2_O_2_-producing lactobacilli also suppress some reproductive tract pathogens [19,20,21,22]. Together, these observations have led to a general belief that H_2_O_2_ production by vaginal lactobacilli provides significant protection against BV-associated bacteria and other reproductive tract pathogens. However, we have recently shown that H_2_O_2_ production by vaginal lactobacilli is implausible as a mechanism of protection *in vivo*, since 1) the hypoxic condition of the vagina precludes bacterial production of H_2_O_2_, 2) the high antioxidant capacity of cervicovaginal fluid (CVF) blocks the microbicidal activity of H_2_O_2_, and 3) H_2_O_2_ is more toxic to vaginal lactobacilli than to the seventeen species of BV-associated bacteria we tested [23,24]. 

Instead of H_2_O_2_, we attribute the broad spectrum, microbicidal protection associated with LMD microbiotas to the production of lactic acid by vaginal lactobacilli. We have previously confirmed that vaginal lactobacilli are the primary source of lactic acid [12,13]. Furthermore, during episodes of BV, lactobacilli and vaginal lactic acid are markedly diminished [25,26,27].

In contrast to H_2_O_2_, 1) lactic acid is produced in the hypoxic environment of the vagina [12,13], 2) CVF does not block the microbicidal activity of lactic acid [24], and 3) *in vitro*, vaginal lactobacilli are unaffected by concentrations of lactic acid that completely inactivate all the BV-associated bacteria we tested [23]. Lactic acid has been shown to inactivate a wide range of other reproductive tract pathogens, including HSV-2 [28], *N. gonorrhoeae* [29], and uropathogenic *Escherichia coli* [30]. In addition to direct inactivation of pathogens, vaginal acidity potentiates the slowing and trapping of HIV-1 virions by cervicovaginal mucus [31,32]. As expected, the extent of protective effect observed in these studies depends on the concentration of lactic acid present. 

Protonated lactic acid (“LAH”) rather than lactate anion (“LA^-^”) is the active, microbicidal form of lactic acid [33,23] and the LAH concentration is a function of both total lactate concentration (total lactate = LAH + LA^-^) and pH or hydrogen ion (H^+^) concentration, as calculated using the pKa of lactic acid (3.86) and the Henderson-Hasselbach equation. LAH concentration increases with increasing total lactate concentration, and/or increasing hydrogen ion concentration (*decreasing* pH). The literature values for ‘normal’ cervicovaginal total lactate and pH, informed by an extensive review of published observations [34] are approximately 0.2% (22 mM) total lactate and pH 4.2. These values correspond to an LAH concentration of approximately 0.06% (7 mM). An LAH concentration this low can only partially inactivate BV-associated bacteria [23], and considerably more LAH is required to inactivate uropathogenic *E. coli* [35], and HIV-1 [33,36,37]. Consequently, based on prior literature values, H_2_O_2_ production by vaginal lactobacilli has been regarded as more important than lactic acid production for protection against reproductive tract pathogens. 

However, we believe that almost all prior studies of “normal” vaginal lactic acid concentration suffered from two major sources of systematic error, both of which underestimate “normal” vaginal total lactate concentration and overestimate “normal” vaginal pH. Hence, prior studies significantly underestimated LAH concentration and, consequently, the microbicidal protection that LAH may provide. 

First, previously reported observations of vaginal lactic acid (measured as total lactate) were almost entirely made without any assessment of the vaginal microbiota. The inclusion criteria were extremely broad, e.g. “regularly menstruating women … [with] no obvious metabolic disturbances” [38], “normal … women of childbearing age” [39]. However, a significant portion of asymptomatic women apparently lack a LMD vaginal microbiota [40,41,42], making it likely that these studies included participants with intermediate or high Nugent scores that are indicative of less protective microbiotas. Indeed, almost all previous studies included participants whose vaginal pH was too high to be compatible with current standards of vaginal health; a vaginal pH greater than 4.5 is now a diagnostic criterion for BV [43], and infection by *T. vaginalis* also causes abnormally high vaginal pH [44,45]. Values above pH 6.5 were reported in many studies, and above pH 8 in more than one. The inclusion of these participants means prior reports of “average” pH were skewed upwards and “average” total lactate concentrations were skewed downwards. 

Second, almost all previous studies ignored the high carbon dioxide (CO_2_) and low oxygen (O_2_) levels that prevail in the vaginal environment. The cervicovaginal partial pressure of CO_2_ is equivalent to systemic levels (38 mm Hg or 5%)[46]; this CO_2_ is rapidly lost when the vaginal lumen or a CVF sample is exposed to air (partial pressure of CO_2_ in air ~ 0.30 mm Hg or 0.04%). Loss of CO_2_ from CVF samples would increase the pH and lead to an overestimate of the *in vivo* value. Furthermore, the cervicovaginal partial pressure of O_2_ is in the hypoxic range of 4-14 mm Hg (2%), with only transient increases due to insertion of a tampon [47] or diaphragm [48], sexual arousal [49] and, presumably, vaginal intercourse. Under hypoxic conditions, vaginal lactobacilli produce primarily lactic acid, but in air (partial pressure O_2_ 160 mm Hg or 21%), many strains of lactobacilli preferentially produce acetic acid [50], and some strains even *consume* previously formed lactic acid to do so [51]. Measurements made in CVF samples collected, processed, and analyzed aerobically, therefore, likely underestimate the concentration of lactic acid present under the hypoxic conditions of the vagina. 

An accurate assessment of the protection that can be provided by vaginal lactic acid therefore requires observations of pH and total lactate concentration that 1) include only participants with an LMD vaginal microbiota, 2) collect CVF samples with minimal aerobic exposure and make measurements under hypoxic conditions, 3) prevent or compensate for the alkalizing effect of CO_2_ loss, and 4) use analysis methods that minimally perturb the biochemical composition of the samples. The data we report here were generated using strict inclusion criteria and a quantitative evaluation of participants’ vaginal microbiotas via Nugent scoring; we measured pH, total lactate and total acetate concentrations in *ex vivo* (freshly collected, undiluted, hypoxically maintained) CVF samples, with prevention of or correction for the effect of CO_2_ loss. In addition, to compare our results with those of earlier investigations, measurements were also made under the aerobic and hypocapnic conditions typical of previous studies. 

## Materials and Methods

### Reagents

Unless otherwise stated, all reagents were supplied by Sigma-Aldrich Inc. (St. Louis, MO).

### Ethical approval

Each participant gave written informed consent at every sample collection; the study and study protocols were approved by the Johns Hopkins University Homewood Institutional Review Board on the Use of Human Subjects.

### Participants

Participants were required to be between 18 and 45 years old, in good general health, at least three days past the end of the most recent menstruation and most recent unprotected penile-vaginal intercourse, at least three weeks past the most recent use of vaginal or systemic antibiotics or antifungals, and free from vaginal symptoms (discharge, odor, itching or discomfort). 

### Collection of cervicovaginal fluid

Cervicovaginal fluid (CVF) was self-sampled at the laboratory using the non-absorbent disposable Softcup™ menstrual device (Evofem Inc., San Diego CA) [52], thereby circumventing the disadvantages of the more common swab or lavage collection methods: namely, exposing the vaginal epithelium to the air with a speculum, as well as high-factor dilution and aeration of the sample in collection or recovery. After vaginal insertion and immediate (~10 sec) removal, the Softcup was quickly placed inside an open 50 mL centrifuge tube and immediately transferred to a nitrogen glove-box that mimicked the hypoxic and hypercapnic condition that prevails in the vagina (partial pressure of O_2_ = 6.0±0.7 mm Hg as measured with an MI-730 oxygen electrode, Microelectrodes Inc., Bedford NH, and partial pressure of CO_2_ = 38±2 mm Hg as analyzed by supplier), or provided a hypoxic and hypocapnic condition (partial pressure of O_2_ = 6.0±0.7 mm Hg in nitrogen without CO_2_, all gases from Airgas Specialty Gases, Radnor PA). There were no transport or storage steps in the protocol; participants inserted and removed the Softcup in a room adjacent to the prepared glove-box, and the cup coated with CVF was transferred to its new environment within seconds. 

### Measurement of pH

The pH of the CVF clinging to the Softcup (still in the open centrifuge tube) was immediately measured inside the glove-box, using an MI-415-6 cm combination pH electrode (Microelectrodes Inc.) designed to measure pH in small volumes or thin layers of fluid, and a digital pH meter (Φ11 pH meter, Beckman Instruments Inc., Fullerton CA). With the possibility of heterogeneity within individual samples, we made three pH measurements in quick succession at different sites on each Softcup; however, the three replicate measurements in each CVF sample never varied by more than ±0.02 pH units, and only the mean of each set of three measurements is reported here. The time elapsing between the initial insertion of the Softcup in the vagina and the completion of all three pH measurements was less than one minute in all cases. 

To observe the time-course of the alkalization caused by loss of CO_2_ from CVF, additional samples (still on the Softcup inside the open centrifuge tube) were placed in an aerobic and hypercapnic incubator (partial pressure of CO_2_ = 38±3 mm Hg in humidified air, as measured by incubator regulator, Thermo Fisher Scientific, Waltham MA) instead of the glove-box, and allowed to equilibrate overnight. Each sample was then brought into room air, the pH microelectrode immediately placed in the sample, and pH monitored over the next ten minutes to determine the time-course of alkalinization due to loss of CO_2_. 

### Sample mass and Nugent scoring

After pH measurement, the centrifuge tube was sealed while still in the glove-box to maintain hypoxic conditions during centrifuging for one minute at 500*g* (IEC-Centra-8 swinging-bucket centrifuge, International Equipment Company, Chattanooga TN) to collect the CVF from the Softcup. The centrifuge tube was returned to the glove-box, opened, and the Softcup removed and discarded. The tube was weighed and the mass of the sample was calculated by reference to the pre-use recorded weight of the tube. After removing sufficient CVF for analysis of total lactate and total acetate concentrations, a sterile cotton swab was dipped in the remaining CVF, rolled out on a microscope slide, and the slide air-dried for Gram-staining and Nugent scoring according to a standard protocol [1]. 

### Measurement of total lactate and total acetate concentrations

Both chromatographic and enzymatic methods were used to measure total lactate and total acetate concentrations. 

Enzymatic analyses used the D-lactic acid/L-lactic acid Enzymatic BioAnalysis/Food Analysis UV method kit and the Acetic acid Enzymatic BioAnalysis/Food Analysis UV method kit (R-Biopharm, Darmstadt, Germany). Unlike the more generally used HPLC method, these enzymatic assays do not require extensive dilution, purification, or other manipulation of the samples. Additionally, the assay reactions and incubations can be carried out in a glove-box, and only removed immediately before the spectrophotometric measurements are made. The time elapsing between removal from our hypoxic glove-box and completion of the spectrophotometric measurements was less than one minute in all cases. 

To mimic procedures used in numerous previous studies, a second cotton swab was dipped in eight CVF samples (in addition to the one used to prepare the microscope slide) and subjected to conditions and processes typical of swabs collected at a clinical site and transported to a laboratory for HPLC analysis. Briefly, each swab-tip was exposed to air and extracted by vortexing in 1.0 mL distilled water. The resulting fluid was centrifuged and syringe-filtered, following [27]. The HPLC analysis was performed using a Lab Alliance Series 1 isocratic system with a 214 nm UV detector (Scientific Systems Inc., State College PA) and a Prevail™ 150x4.6 mm organic acid column (Alltech Associates Inc., Deerfield IL). Data were collected and analyzed using the Peaksimple chromatography data system and software (SRI Instruments, Torrance CA). To compare methods, an aliquot of each HPLC-prepared fluid was also analyzed using the enzymatic method. 

A set of known-concentration standard solutions of acetate and lactate were prepared (using sodium acetate and sodium lactate, to avoid errors due to polymerization of concentrated lactic acid that persists for considerable periods after dilution). These concentration standards were analyzed in control experiments and in association with every CVF sample to ensure consistency and comparability of data collected by different methods or under different conditions. 

CVF (for enzymatic analysis) and CVF extracts (for HPLC analysis) were serially diluted with clinical saline and the entire series assayed, guaranteeing at least two data points falling within the dynamic range of each method for every participant-sample. Correcting for the individual dilution-factors within the series typically yielded similar but not identical values for the original sample-concentration; the reported values for original sample-concentrations are therefore the mean of at least two data points falling within the dynamic range of the indicated method.

### Statistical analysis

Results are reported as mean ± standard deviation of at least eight independently repeated observations (each observation using a single sample, and with at least two replicate measurements made on each sample). For calculating means, samples with undetectable analytes were assigned values equal to half the detection threshold. The difference between two means was tested using a two-tailed Student’s *t* test (comparisons were unpaired unless otherwise indicated); *p* values ≤ 0.05 were considered to be statistically significant. Statistical analysis was performed using PHStat2 version 3.0 (Microsoft Excel add-on). 

## Results

A total of 59 participants were recruited, primarily from among the students and staff of the university; they were between 19 and 44 years old (mean age 23±6 years), and self-identified as non-Hispanic White (*n* = 23), Black (*n* = 17), or Asian (*n* = 19). In all, 67 CVF samples were collected; three samples were excluded due to Nugent score >3, and one due to leucorrhea (>10 polymorphonuclear leukocytes per high powered field) [53]. The low rate of excluded samples observed is consistent with the low BV-risk composition of the study population and the strict requirements for participation.

### Cervicovaginal pH

**Figure 1:**
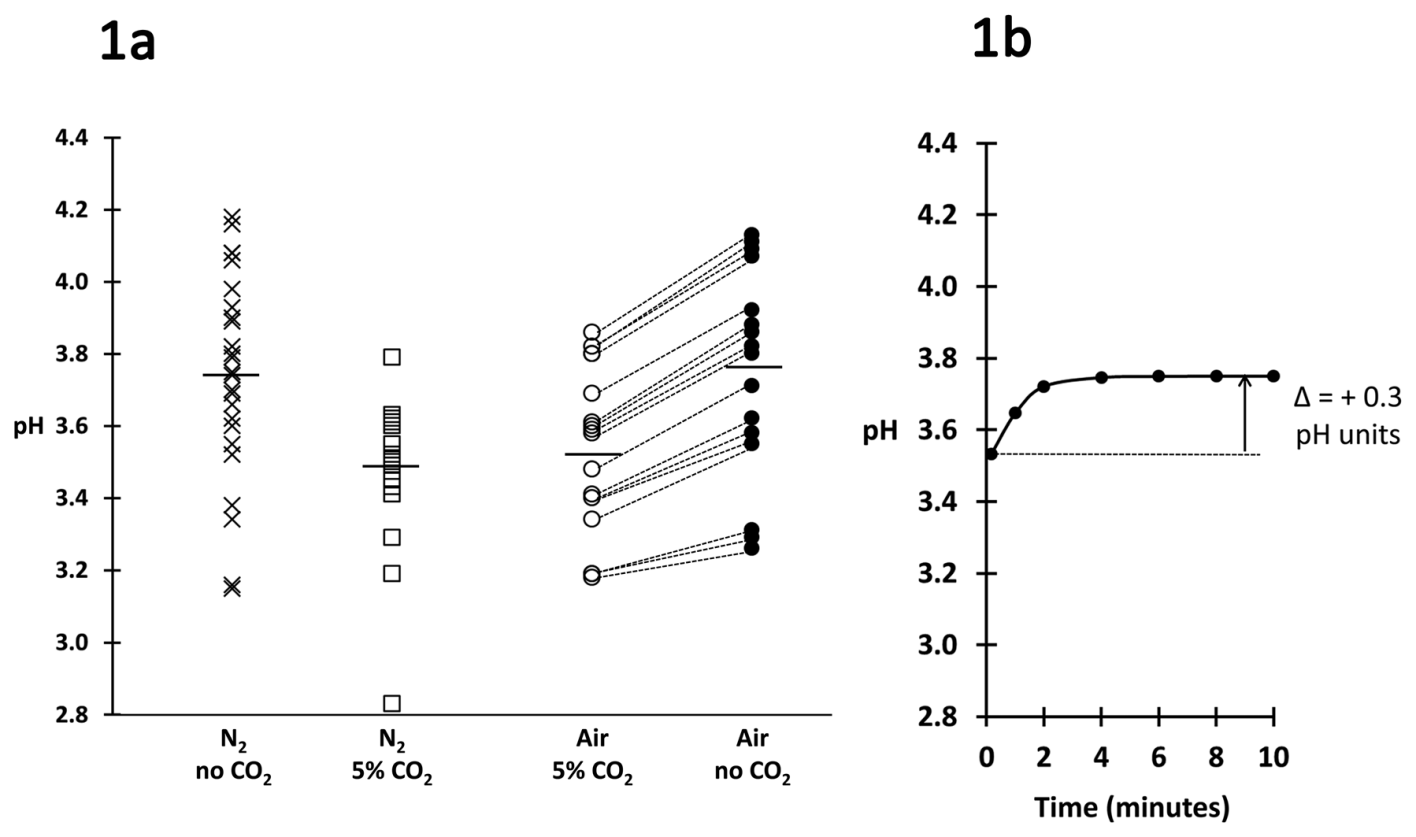
A: Effects of CO_2_ on pH. First column: *X* *plot* *symbols* (*n* = 24) indicate the pH of samples measured in N_2_ without CO_2_. Second column: *open* *squares* (*n* = 24) indicate the pH of samples measured in N_2_ with 5% CO_2_. Third column: *open* *circles* (*n* = 16) indicate the pH of samples incubated overnight in air with 5% CO_2_ and measured immediately after exposing to air. Dotted lines link the values of these samples to *filled* *circles* in the fourth column, which indicate the pH of the same samples two minutes after exposing to air. A horizontal bar indicates the mean value for each column. **B**: Time course of increase in mean pH of the 16 samples that had been equilibrated in air with 5% CO2 (shown in Figure 1A, third column) following exposure to air.

The pH of 24 CVF samples was measured hypoxically and hypocapnically (low O_2_ and CO_2_); the mean pH was 3.74±0.29 (range 3.15-4.18) (Figure 1A, X plot symbols). Another 24 samples were measured hypoxically and hypercapnically (low O_2_ and ~ 5% CO_2_ in N_2_), mimicking the vaginal environment, and the mean pH was significantly lower at 3.48±0.18 (range 2.83-3.79, *p* = 0.03) (Figure 1A, open squares). To confirm that this reduction in pH was due to CO_2_, and also to determine the time-course of alkalization caused by loss of CO_2_, 16 additional samples were equilibrated aerobically and hypercapnically (~5% CO_2_ in humidified air). Each sample was then exposed to ambient air and the pH monitored over time. The mean initial pH (measured after ~10 sec exposure) was 3.53±0.23 (Figure 1A, open circles, and initial data point in Figure 1B), also significantly lower than the mean of the samples measured in the hypoxic and hypocapnic condition (*p* = 0.01) but not significantly different from the mean of the samples measured in the hypoxic and isocapnic condition. The pH of the samples exposed to air increased by approximately 0.3 pH units within 3 minutes (Figure 1A, filled circles), and reached a final mean pH of 3.75±0.19 after 10 minutes, a value almost identical to that of the samples measured in the hypoxic and hypocapnic condition (Figure 1B). 

### Total lactate and total acetate concentrations

Calibration experiments using known-concentration standard solutions of lactate and acetate showed that the detection threshold of the enzymatic method was 0.0007% for lactate and 0.0003% for acetate; the lower limit of accurate quantification for the enzymatic method was 0.035% for lactate and 0.038% for acetate. The detection threshold for the HPLC method was 0.0027% for lactate and 0.0025% for acetate, while the lower limit of accurate quantification was 0.37% for lactate and 0.41% for acetate (data not shown). 

Control experiments using the standard lactate and acetate solutions showed the enzymatic method was not affected by aerobic versus hypoxic or isocapnic versus hypocapnic conditions (data not shown). To rule out possible interference in the enzymatic method, several CVF samples were serially diluted with clinical saline and a known, constant amount of lactate and acetate added to all the dilutions. The dilutions were then analyzed by the enzymatic method; all dilutions showed a constant and appropriate increase in signal, compared to dilutions without added lactate or acetate (data not shown).

The mean total lactate concentration of 48 freshly collected, hypoxically maintained CVF samples analyzed enzymatically was 1.0±0.2% (111±22 mM), range 0.72-1.48%. In contrast, acetate was undetectable (detection threshold of assay 0.0003%) in 44 samples by enzymatic assay. The four samples with detectable acetate ranged between 0.0025% and 0.028%.

**Figure 2:**
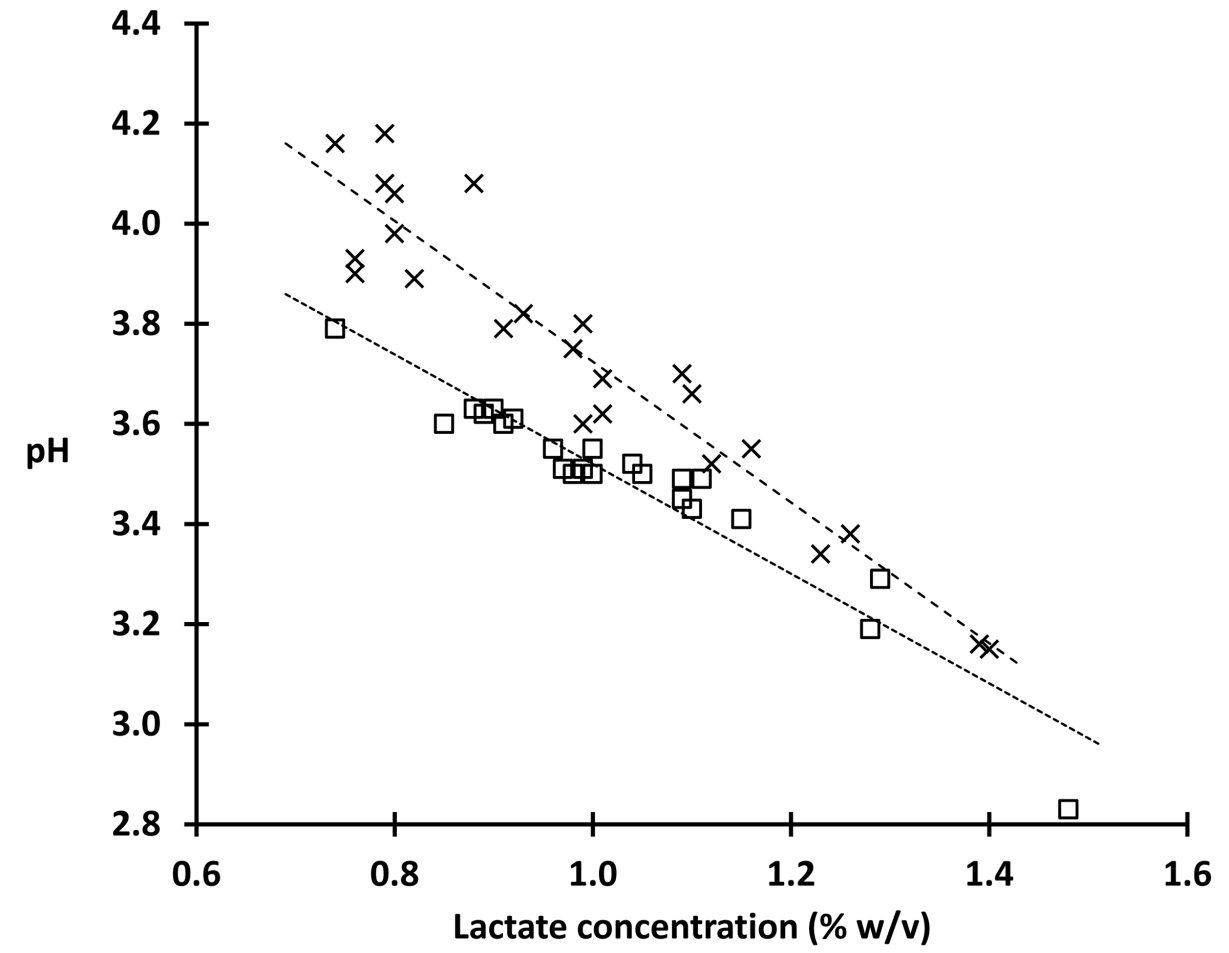
Vaginal pH is tightly correlated with total lactate concentration: total lactate concentration in 48 fresh CVF samples, versus pH measured in 100% N_2_ (X plot symbols) or 95%N_2_ with 5% CO_2_ (open squares); *r* ^2^ = 0.91 for both trend lines (least squares fit lines).

We found a strong inverse linear relationship between the total lactate concentration and the pH of CVF (Figure 2: least squares fit for closed circles: pH = -1.1 x total lactic acid concentration (%) + 4.6; *R* ^2^ = 0.91), further supporting lactic acid as the primary determinant of vaginal acidity when lactobacilli dominate the vaginal microbiota. The tight correlation we observe between total lactate concentration and pH also indicates that the permeability ratio of the vaginal epithelium for lactate and hydrogen ions is similar among individuals. We note that in Figure 2 at the lower total lactate concentrations there was a larger increase in pH with loss of CO_2_ (open circles vs filled circles) compared to the lesser increase in pH observed at higher total lactate concentration, presumably since buffer capacity is proportional to total lactate. In addition, we found no correlation between sample pH or total lactate concentration with sample mass, or with Nugent score (within the studied 0-3 range), and there was no significant difference between the means of total lactate measured with or without CO_2_ (1.03 and 0.99% respectively, P = 0.4). 

Eight additional CVF samples (mean total lactate 1.04±0.20% by enzymatic analysis) were processed for HPLC analysis. Processing samples for HPLC significantly decreased total lactate to 0.63±0.23% (*p* = 0.001) when assayed both by HPLC and by the enzymatic method. Moreover, acetate was detectable in only one of eight samples by enzymatic analysis *before* preparation for HPLC but was measurable in seven of these eight samples after preparation for HPLC with a mean concentration of 0.17±0.09%. Preparation of samples for HPLC analysis caused a greater than twenty-fold increase in acetic acid concentration to levels consistent with those reported in the prior literature. 

## Discussion

Almost all previously reported observations of vaginal pH and total lactate concentration were made without assessing the vaginal microbiota, and without controlling or correcting for the changes that occur when CVF is removed from the vaginal environment and prepared for analysis. The present study includes only participants with a lactobacillus-morphotype-dominated (LMD) vaginal microbiota, and reports measurements made in CVF samples collected without dilution or other modification, with rigorously minimized exposure to air, as well as ameliorating and correcting for the effect of CO_2_ loss.

The mean pH of 24 samples measured under hypoxic and hypercapnic conditions mimicking those of the vagina was 3.5 ± 0.2; this is 0.7 pH units below the reviewed literature value for healthy vaginal pH of 4.2 [34], a difference that corresponds to a five-fold higher hydrogen ion concentration. Comparing our hypocapnic and hypercapnic measurements indicates that approximately 0.3 pH units of this difference can be attributed to our maintaining the CO_2_ levels in samples during pH measurements (Figure 1A), an interpretation further supported by the time course data on loss of CO_2_ after CVF was removed from a 5% CO_2_ environment (Figure 1B). We attribute the remaining 0.4 pH units decrease we attribute to the restriction of the data to samples with LMD microbiota. 

The mean total lactate concentration we measured with the minimally disruptive enzymatic method under hypoxic conditions was 1.0% ± 0.2%, again about five-fold higher than the prior reported values. Comparing the results of eight samples assayed by both the enzymatic and HPLC methods indicates that slightly more than half this difference is caused by preparation of samples for HPLC analysis. Similarly we attribute prior observations of acetic acid concentrations were artifactually increased during sample preparation for analysis. Finally, we attribute the remainder of the differences to the restriction of the study to samples with an LMD microbiota.

Together, the higher concentrations of hydrogen ions and total lactate that we determined here indicate an 11-fold higher concentration of protonated lactic acid, LAH: based on the pKa of lactic acid and the Henderson Hasselbalch relation, the mean LAH concentration in our samples was 0.69% (76 mM), compared to an LAH concentration of 0.06% (7 mM) when calculated from the literature values of vaginal pH and total lactate concentration. This is a critical difference in terms of microbicidal activity, since we have shown that it is only LAH that inactivates HIV, with the lactate anion (LA^-^) being totally inactive [33]. The low concentration of LAH indicated by the data in the prior literature has only modest inhibitory effects against BV-associated bacteria in vitro [23], and had little or no effect on 5 of 6 clades of HIV [33]. In contrast, the eleven-fold higher concentration of LAH reported here caused complete inactivation of all seventeen species of BV-associated bacteria tested [23] and rapidly inactivated HIV by more than 1000-fold [33]. Therefore, the *in vivo* microbicidal action of vaginal lactic acid when lactobacilli dominate the vaginal microbiota may be significantly more protective than previously thought. Although an ejaculate transiently alkalinizes the vagina after unprotected intercourse, reducing the protective actions of vaginal acidity against male-to-female transmission, the level of vaginal lactic acid we document here is likely to reduce female-to-male transmission of vaginally shed acid-sensitive pathogens. 

Unfortunately, a significant number of women experience the transient, often recurrent loss of the LMD vaginal microbiota and its replacement by Gram-negative and Gram-variable bacteria (bacterial vaginosis), and probiotic strains of lactobacilli are being developed that might enable these women to maintain an LMD vaginal microbiota. Currently, the ability to produce H_2_O_2_ is the most widely applied criterion for selecting probiotic strains, but our results suggest it may be more relevant to select strains for their ability to produce high lactate concentration and low pH. Our results also indicate the need to assess the stability and efficacy of microbicidal agents and other vaginal pharmaceuticals in this low pH and high lactic acid range. 
